# A Machine Learning Model Based on microRNAs for the Diagnosis of Essential Hypertension

**DOI:** 10.3390/ncrna9060064

**Published:** 2023-10-25

**Authors:** Amela Jusic, Inela Junuzovic, Ahmed Hujdurovic, Lu Zhang, Mélanie Vausort, Yvan Devaux

**Affiliations:** 1 Cardiovascular Research Unit, Department of Precision Health, Luxembourg Institute of Health, L-1445 Strassen, Luxembourg; 2 HAYA Therapeutics SA, Route De La Corniche 6, SuperLab Suisse—Batiment Serine, 1066 Epalinges, Switzerland; 3 Department of Internal Medicine, Medical Center “Plava Medical Group”, Mihajla i Živka Crnogorčevića do br. 10, 75000 Tuzla, Bosnia and Herzegovina; 4 Bioinformatics Platform, Luxembourg Institute of Health, L-1445 Strassen, Luxembourg

**Keywords:** miRNA, biomarkers, hypertension, machine learning

## Abstract

Introduction: Hypertension is a major and modifiable risk factor for cardiovascular diseases. Essential, primary, or idiopathic hypertension accounts for 90–95% of all cases. Identifying novel biomarkers specific to essential hypertension may help in understanding pathophysiological pathways and developing personalized treatments. We tested whether the integration of circulating microRNAs (miRNAs) and clinical risk factors via machine learning modeling may provide useful information and novel tools for essential hypertension diagnosis and management. Materials and methods: In total, 174 participants were enrolled in the present observational case–control study, among which, there were 89 patients with essential hypertension and 85 controls. A discovery phase was conducted using small RNA sequencing in whole blood samples obtained from age- and sex-matched hypertension patients (n = 30) and controls (n = 30). A validation phase using RT-qPCR involved the remaining 114 participants. For machine learning, 170 participants with complete data were used to generate and evaluate the classification model. Results: Small RNA sequencing identified seven miRNAs downregulated in hypertensive patients as compared with controls in the discovery group, of which six were confirmed with RT-qPCR. In the validation group, miR-210-3p/361-3p/362-5p/378a-5p/501-5p were also downregulated in hypertensive patients. A machine learning support vector machine (SVM) model including clinical risk factors (sex, BMI, alcohol use, current smoker, and hypertension family history), miR-361-3p, and miR-501-5p was able to classify hypertension patients in a test dataset with an AUC of 0.90, a balanced accuracy of 0.87, a sensitivity of 0.83, and a specificity of 0.91. While five miRNAs exhibited substantial downregulation in hypertension patients, only miR-361-3p and miR-501-5p, alongside clinical risk factors, were consistently chosen in at least eight out of ten sub-training sets within the SVM model. Conclusions: This study highlights the potential significance of miRNA-based biomarkers in deepening our understanding of hypertension’s pathophysiology and in personalizing treatment strategies. The strong performance of the SVM model highlights its potential as a valuable asset for diagnosing and managing essential hypertension. The model remains to be extensively validated in independent patient cohorts before evaluating its added value in a clinical setting.

## 1. Introduction

Hypertension is a complex disease resulting from dynamic interactions between genetic, lifestyle, and environmental factors. Given multifaceted pathogenesis, the etiology of hypertension is unknown in 90–95% of cases and is referred to as essential hypertension. Recent comprehensive analyses of hypertension prevalence have shown that the number of people aged 30–79 years with hypertension doubled from 1990 to 2019, with most of the increase occurring in low-income and middle-income regions [1]. According to the World Health Organization, 46% of adults with hypertension are unaware that they have this condition (World Health Organization). Managing hypertension remains a significant challenge for public health systems. Despite advancements in blood pressure measurement methods and the availability of effective and safe antihypertensive medications, a considerable number of individuals with hypertension go undiagnosed, and many of those receiving treatment do not achieve optimal blood pressure control. For instance, Huguet et al. (2021) reported a prevalence of 37% of undiagnosed hypertension patients in the clinical trial NCT0354576 [2]. Another study investigating undiagnosed hypertension in emergency departments in the United Kingdom reported approximately 3% to 15% of asymptomatic adults, with about 50% having Stage 1 hypertension, 25% to 36% with Stage 2 hypertension, and 12% to 30% with Stage 3 hypertension [3]. Undiagnosed hypertension refers to an average blood pressure level that exceeds the criteria for diagnosis without having any prior ICD-10 codes (the International Classification of Diseases, 10th Edition) indicating the presence of hypertension [3]. Of note, ICD-10 codes are alphanumeric codes used by healthcare professionals and medical coders to categorize and document various health conditions, diseases, injuries, and other medical diagnoses. In addition, a significant proportion of hypertension patients are untreated with antihypertensive therapy. Since asymptomatic hypertension remains undiagnosed and untreated, it can have serious consequences, leading to severe organ damage and increasing the rate of cardiovascular diseases and premature death [4]. In line with epidemiological data, hypertension remains a major risk factor in stroke, coronary artery disease, renal disease, heart failure [5,6], and peripheral vascular disease [2], which, together, constitute the first cause of mortality and represent a high socioeconomic burden.

The current diagnostic procedure for essential hypertension involves multiple blood pressure measurements taken over time, as well as a review of the patient’s medical history, symptoms, and risk factors. However, this approach is not always effective in identifying patients with essential hypertension, as many people with the condition may have normal blood pressure readings or do not present with any symptoms. In addition, blood pressure readings can vary significantly throughout the day and in different settings, posing challenges in obtaining precise measurements. Additionally, the phenomenon of white coat hypertension, where stress or anxiety leads to temporarily elevated blood pressure in clinical settings, can yield inaccurate readings. Conversely, individuals with masked hypertension have normal clinical blood pressure but experience elevated levels in their daily lives, often leading to under-diagnosis. Furthermore, some patients exhibit resistance to standard antihypertensive treatments, making effective management challenging. Lastly, high blood pressure can be linked to underlying medical conditions or medications, occasionally escaping detection during the diagnostic process.

Despite an improvement in hypertension awareness, straightforward diagnosis of hypertension with a sphygmomanometer, and relatively easy treatment with low-cost drugs, recent data on hypertension prevalence has pinpointed that significant gaps in diagnosis and treatment remain. This indicates that alternative and creative strategies must be explored to enhance the detection and treatment of this condition. For instance, discovering novel, clinically applicable biomarkers predicting blood pressure (BP) elevation may shed some light on the molecular mechanism involved in hypertension development and would allow for the improvement of primary prevention and a reduction in morbidity and mortality rates via tailored treatments.

Noncoding RNAs, and especially microRNAs (miRNAs), regulate gene expression and are involved in age-related cardiovascular disease [7]. Circulating in the bloodstream, miRNAs appeared as attractive biomarkers for many cardiovascular diseases, including essential hypertension [8,9,10]. For instance, decreased levels of cardiac-enriched miR-133a are associated with the development of left ventricular hypertrophy in patients with arterial hypertension [11]. Several circulating miRNAs (miR-145, miR-4516, miR-1299, and miR-30a-5p) are regulated in primary hypertension [12,13,14]. Out of 21 deregulated circulating miRNAs in hypertensive patients, 4 of them are associated with albuminuria, indicating renal damage [15]. Circulating levels of miR-9 and miR-126 are regulated in essential hypertension and are associated with prognosis [16]. Overall, since miRNAs participate in the development and progression of hypertension [17] and are regulated upon antihypertensive therapy [18,19,20], they represent promising diagnostic, prognostic, and surrogate end-point biomarkers for the follow-up of hypertension control. The incorporation of miRNA expression profiles alongside conventional blood pressure measurements in clinical practice has the potential to enrich our comprehension and management of hypertension. miRNA expression profiles can enhance traditional blood pressure readings by enabling early detection, refining risk stratification, tailoring treatments, monitoring the effectiveness of antihypertensive therapies, and deepening our understanding of hypertension’s pathophysiology, as well as predicting treatment responses. The synergy of miRNA data with blood pressure measurements may lead to more precise risk evaluations, with specific miRNA patterns linked to an elevated risk of cardiovascular events, thereby assisting in the identification of individuals who may benefit from intensified monitoring or more aggressive treatment strategies.

Artificial intelligence (AI) is an evolving cluster of interrelated fields, including machine learning (ML), the most prominent [21]. Current progress in precision medicine approaches using ML-based models integrating high-throughput multi-omics data and clinical risk factors shows outstanding potential to improve clinical strategies for a better understanding and treatment of hypertension. ML-based techniques have been applied in high blood pressure studies based on hypertension stage classifications using clinical data and blood pressure estimation based on related physiological signals, demonstrating an improvement in hypertension prediction, diagnosis, and classification [22,23,24]. A random forest model relying on clinical data and electrocardiogram (ECG)-derived features distinguished hypertensive from normotensive patients with 84.2% accuracy, 78.0% specificity, and 84.0% sensitivity [25]. Recently, the potential added value of circulating miRNAs in ML models aiming to stratify cardiovascular risk in patients with end-stage renal disease was reported [26]. Using multiple ML feature selection approaches, miR-636 and miR-187-5p were found to discriminate patients with pulmonary arterial hypertension from healthy controls [27]. However, despite the potential of circulating miRNAs to be used as biomarkers for essential hypertension, available data on ML-based approaches remain limited.

In the present study, we assessed whether an ML-based model integrating circulating miRNA expression patterns and clinical risk factors could improve essential hypertension risk prediction above the current diagnostic procedure, which relies primarily on blood pressure measurements and medical history.

## 2. Results

### 2.1. Study Participants

Of the 193 participants eligible for the present study, 174 subjects with RNA samples reaching quality and quantity criteria (cf. Section 5) were included. In total, 89 participants had hypertension (systolic/diastolic blood pressure >140/90 mmHg), and 85 participants with systolic/diastolic blood pressure <140/90 mmHg were classified as control subjects. In total, 60 subjects were included in a discovery phase with small RNA-sequencing, and the remaining 114 subjects were included in a validation step using RT-qPCR. Clinical and demographic features of the study population are summarized in Table 1. In both discovery and validation cohorts, participants in the hypertension group had higher BMI and systolic and diastolic blood pressure and were more often smokers as compared with the control group. A family history of hypertension was more frequent in the hypertension group as compared with the control group in the discovery cohort. Finally, there were fewer patients consuming alcohol in the hypertension group as compared with the control group in the validation cohort. Hypertension subjects and controls in the discovery cohort were age- and sex-matched, and there was a balanced number of males and females of comparable age in both groups of the validation cohort.

### 2.2. Discovery Phase

**Figure 1:**
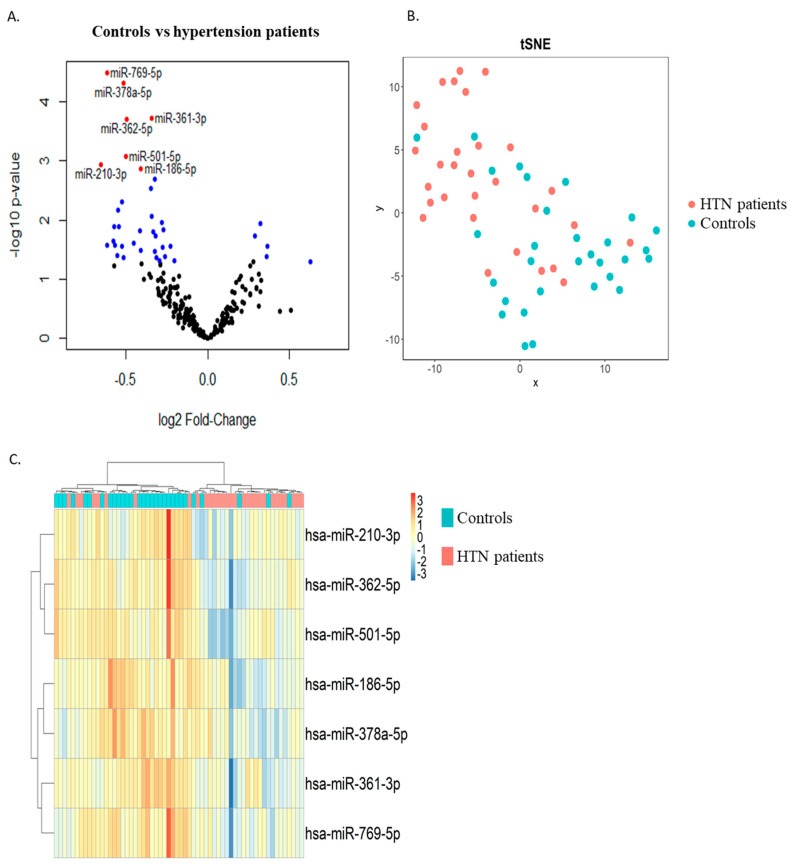
Discovery study. RNAs extracted from whole blood samples of 2 age- and sex-matched groups of hypertension and control subjects were subjected to small RNA sequencing. (**A**) Volcano plot showing differentially expressed miRNAs between hypertension patients and controls. Six miRNAs were upregulated and thirty-four miRNAs were downregulated in the hypertension group as compared with the control group with a *p*-value < 0.05 (blue dots) and a *p*-value > 0.05 (black dots). Of these, 7 miRNAs were downregulated in the hypertension group with an FDR < 0.05 (red dots). (**B**) t-distributed stochastic neighbor embedding (t-SNE) showing the distribution of the samples using the 7 miRNAs with FDR < 0.05 (blue dots: control subjects, orange dots: hypertension subjects). (**C**) Heatmap showing unsupervised hierarchical clustering using the 7 miRNAs with FDR < 0.05 (blue: control subjects, orange: hypertension subjects). HTN—hypertension.

Two groups of thirty age- and sex-matched hypertension and control subjects were included in the discovery phase. RNAs extracted from whole blood samples collected at study enrolment were used as input for small RNA sequencing. Differential expression analysis allowed for the identification of 6 miRNAs upregulated and 34 miRNAs downregulated in the hypertension group as compared with the control group with a *p*-value < 0.05 (blue dots in Figure 1A). Among these, seven miRNAs were downregulated in the hypertension group with an FDR < 0.05 (red dots in Figure 1A and Table 2). These seven miRNAs were able to discriminate, with a relatively good capacity, hypertension patients from controls, as shown by the t-distributed stochastic neighbor embedding (t-SNE) clustering technique (Figure 1B) and via unsupervised hierarchical clustering (Figure 1C).

### 2.3. Replication Phase

**Figure 2:**
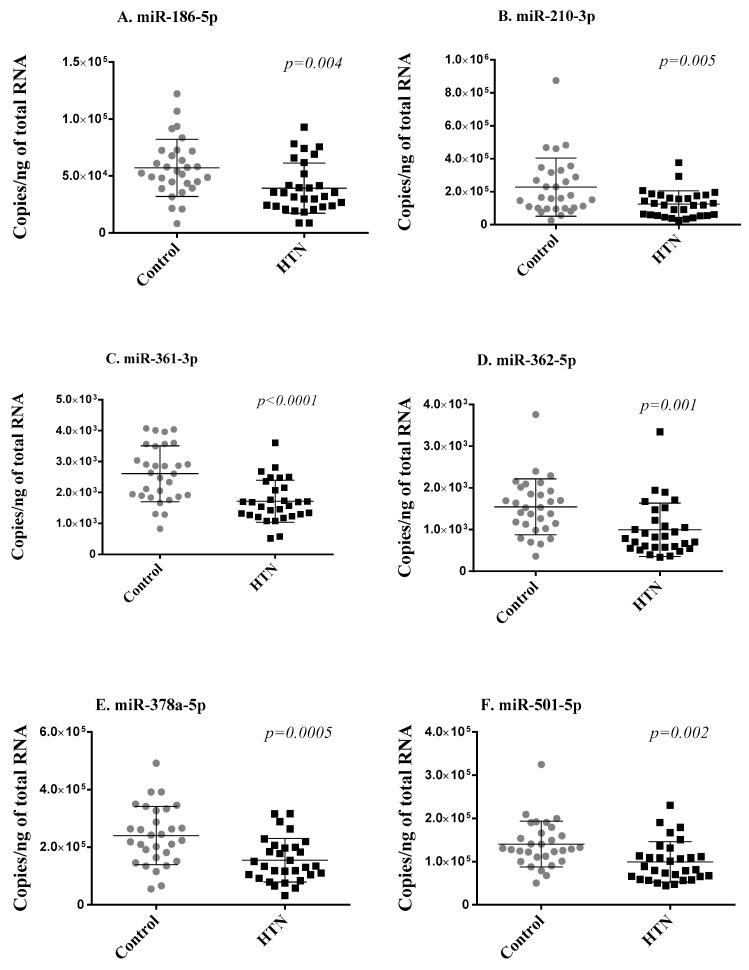
Replication phase. Six miRNAs (**A**–**F**) differentially expressed in small RNA sequencing were assessed via RT-qPCR in whole blood samples of the 2 groups of 30 age- and sex-matched hypertension subjects and 30 control subjects forming the discovery cohort. Expression levels of miRNAs are expressed as the number of copies per ng of RNA and are shown as scatterplots indicating mean ± standard deviation. *p*-values from Mann–Whitney Rank Sum test are indicated.

To address the robustness of small RNA sequencing results, we first measured the seven differentially expressed miRNAs using RT-qPCR in the two groups of 30 age- and sex-matched hypertension subjects and controls from the discovery cohort. Of the seven miRNAs, miR-769-5p showed Cq values > 32 in more than 80% of samples and was discarded from further analysis for unreliable assessment. The expression levels of the six miRNAs reliably measured with RT-qPCR (miR-186-5p, miR-210-3p, miR-361-3p, miR-362-5p, miR-378a-5p, miR-501-5p) significantly correlated with sequencing data (Supplementary Figure S2) and were consistently downregulated in the hypertension group as compared with the control group, thereby replicating the small RNA sequencing results (Figure 2).

### 2.4. Validation Phase

**Figure 3:**
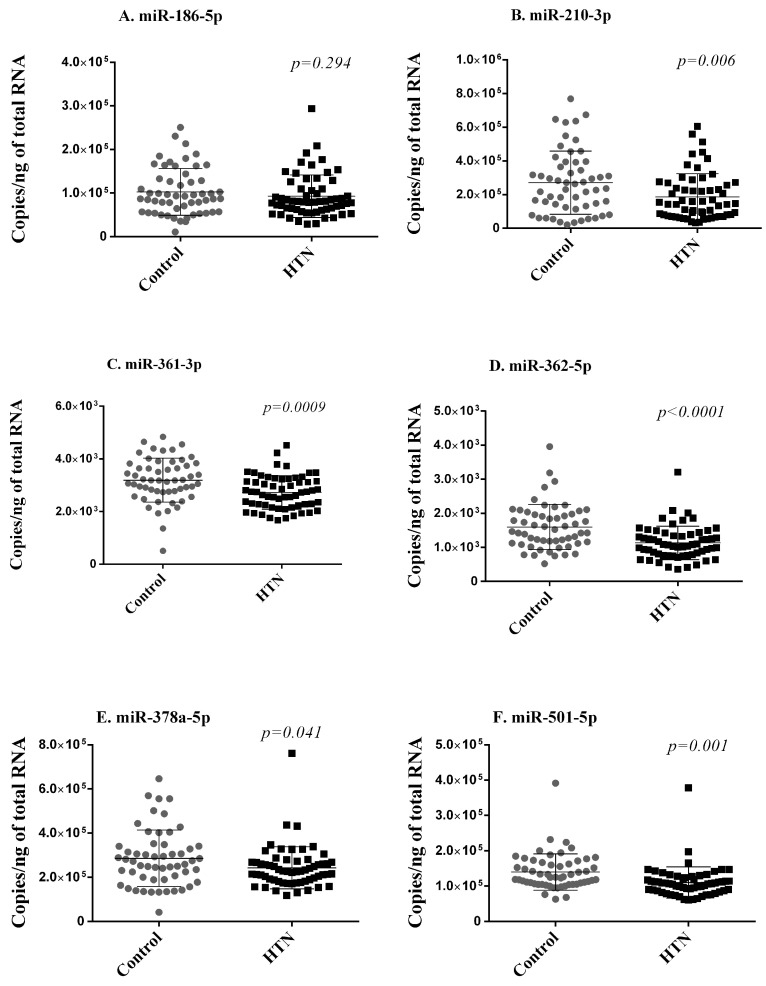
Validation phase. Six miRNAs (**A**–**F**) differentially expressed in small RNA sequencing and replicated in the discovery cohort were assessed with RT-qPCR in whole blood samples of 59 hypertension subjects and 55 controls of the validation cohort. Expression levels of miRNAs are expressed as the number of copies per ng of RNA and are shown as scatterplots indicating mean ± standard deviation. *p*-values from the Mann–Whitney Rank Sum test are indicated.

Next, using RT-qPCR, we quantified the expression levels of the six miRNAs differentially expressed in small RNA sequencing and replicated in the discovery cohort in the whole blood samples of 59 hypertension subjects and 55 controls of the validation cohort. These 114 participants are independent of the 60 participants included in the discovery cohort. To keep the test data completely unseen for the evaluation of the machine learning model, we compared the expression of the miRNAs between the controls (44) and hypertension subjects (47) from 91 participants after excluding the 23 participants in the test dataset. Five of these six miRNAs (miR-210-3p, miR-361-3p, miR-362-5p, miR-378a-5p, miR-501-5p) were less expressed in hypertension subjects as compared with controls, thereby validating, in an independent cohort, their downregulation in subjects with hypertension (Figure 3).

### 2.5. Effect of Medication

To test the potential effect of medication on the regulation of miRNAs between hypertension subjects and controls, we compared the expression levels of six miRNAs (miR-186-5p, miR-210-3p, miR-361-3p, miR-362-5p, miR-378a-5p, miR-501-5p) between the controls, hypertension subjects not receiving antihypertensive treatment, and hypertension subjects receiving antihypertensive treatment. We did not find consistent associations between miRNA levels and medication in either the discovery or validation cohorts, apart from a lower level of miR-361-3p in treated hypertension subjects as compared with untreated hypertension subjects in the validation cohort, which was not confirmed in the discovery cohort (Supplementary Figures S3 and S4). This observation supports the apparent absence of the effect of antihypertensive medication on miRNA levels in the present study population.

### 2.6. Correlation between miRNA Levels and Clinical Features

We then addressed the correlation between the expression levels of six miRNAs (miR-186-5p, miR-210-3p, miR-361-3p, miR-362-5p, miR-378a-5p, miR-501-5p) determined with RT-qPCR in whole blood samples of the 55 controls and 59 hypertension subjects and clinical characteristics. We observed weak-to-moderate negative correlations between all six miRNAs except miR-186-5p and BMI, SBP, and DBP. None of the tested miRNAs correlated with age. Overall, miRNA expression levels tended to positively correlate with each other, and strong correlations were observed between miR-361-3p and miR-186-5p (r = 0.77, *p* < 0.0001) and between miR-378a-5p and miR-501-5p (r = 0.85, *p* < 0.0001) (Supplementary Figure S5).

### 2.7. Machine Learning Model for Hypertension Prediction

Seven of the most common risk factors of hypertension (age, gender, BMI, current smoker, former smoker, alcohol use, and hypertension family history [22]) and five miRNAs consistently downregulated in hypertension subjects as compared with controls (miR-210-3p, miR-361-3p, miR-362-5p, miR-378a-5p, miR-501-5p) were used to build an ML model of hypertension. Continuous variables were transformed using cube root since log2 transformation made some variables highly left-skewed. Categorical variables were one-hot encoded, i.e., created a binary column for each category. The ML workflow is described in Supplementary Figure S1. Of the 112 subjects with complete data from the validation cohort, 20% (n = 23) were randomly extracted as the test dataset, which was used only for model evaluation. The remaining samples (n = 89) from the validation cohort, combined with the sequenced samples with complete clinical data (n = 58), were used as training datasets for feature selection and hyperparameter tuning. According to the analysis of Recursive Feature Elimination with 10CV using four different estimators, the optimal number of features was set to seven, and logistic regression was selected as the optimal estimator, which provided the highest balanced accuracy of 0.83 (Supplementary Figure S6). We then performed recursive feature elimination with logistic regression in 10 sub-training sets split by a 10CV applied to the training dataset (n = 147). The features sex, BMI, current smoker, alcohol use, hypertension family history, miR-361-3p, and miR-501-5p were selected at least eight times in the ten sub-training sets (Supplementary Table S3).

After the hyperparameter tuning of six different classifiers using the training data of the seven selected features, the support vector machine (SVM) classifier provided the highest mean balanced accuracy with two repeated 10CV, and the difference between the training and validation scores was 5% of the training score (Supplementary Table S4). On the test dataset, the SVM model had an AUC of 0.9, a balanced accuracy of 0.87, a precision of 0.9, a sensitivity of 0.83, a specificity of 0.9, and an F1 score of 0.87 (Table 3). Considering the bias that the train–test–split may produce, we finally tested the model on the entire dataset of 174 subjects using Leave-One-Out Cross-Validation (LOOCV), and we obtained an AUC of 0.89, a balanced accuracy of 0.83, a precision of 0.87, a sensitivity of 0.8, a specificity of 0.86, and an F1 score of 0.83 (Table 3 and Supplementary Figure S7A,B).

We then evaluated the performance of the SVM model only with the five clinical variables from the selected features. On the test dataset, the model had an AUC of 0.89, a balanced accuracy of 0.87, a precision of 0.9, a sensitivity of 0.83, a specificity of 0.9, and an F1 score of 0.87 (Table 3). In the entire dataset of 174 subjects using LOOCV, we obtained an AUC of 0.87, a balanced accuracy of 0.79, a precision of 0.81, a sensitivity of 0.79, a specificity of 0.8, and an F1 score of 0.8 (Table 4 and Supplementary Figure S8). We then compared the scores of the model with seven selected features and five clinical features, only estimated from each 10-fold repeated 10CV using Student’s *t*-test. We found that AUC, balanced accuracy, precision, and specificity were significantly (*p* < 0.05) decreased in the model with a clinical feature only, while the F1 score was decreased with *p* = 0.077, and sensitivity was unchanged (Supplementary Figure S7C,D).

## 3. Discussion

Emphasizing the need for novel biomarkers of essential hypertension, we employed machine learning to construct a classification model distinguishing hypertensive patients from non-hypertensive controls. Our model incorporated five clinical parameters (gender, BMI, smoking status, alcohol consumption, family history of hypertension) and two miRNAs (miR-361-3p and miR-501-5p). This model demonstrated performance levels suitable for clinical applicability, with an AUC of 0.90. Of the five miRNAs consistently associated with hypertension in the present study, miR-210-3p, miR-361-3p, miR-378a-5p, and miR-501-5p have not been previously shown to be associated with essential hypertension.

Non-coding RNAs (ncRNAs), which encompass different categories like miRNAs, lncRNAs, and circular RNAs (circRNAs), hold significant relevance in clinical applications. Recent research has unveiled that multiple types of ncRNAs play a role in controlling vascular tone to influence the pathophysiology of arterial hypertension (AH) [28]. For instance, lncRNAs contribute to the regulation of vascular smooth muscle cell function, and miR-221 and miR-222 impact lnc-Ang362, which, in turn, affects the growth of vascular smooth muscle cells [29]. Several lncRNAs, namely, NR_027032, NR_034083, and NR_104181, can serve as biomarkers for diagnosing AH [29]. Another lncRNA, GAS5 (growth arrest-specific 5) plays a role in regulating vascular remodeling in hypertension, with primary expression in endothelial cells (ECs) and VSMCs [28]. Several circRNAs appear to be associated with vascular endothelial dysfunction, which may contribute to the development of arterial hypertension. CircRNAs 0037911 and 0126991 exhibit significant upregulation in the blood of hypertension patients, while circRNA 0005870 shows downregulation [30]. These two circRNAs appear to be promising potential biomarkers for hypertension. Many research studies have explored the role of miRNAs in controlling the function of endothelial cells during the process of angiogenesis. The involvement of miRNAs in the restenosis process has been substantiated through the detection of several miRNAs (such as miR-21, miR-146, and miR-142-3p) showing abnormal expression in stented arteries in pig models [31]. The findings of Parthenakis et al., 2016, underscore the importance of miR-21 in the process of vascular remodeling and suggest its potential use as a prognostic marker and a target for therapeutic interventions. Low levels of miR-21 are closely linked to an improvement in arterial stiffness in individuals with well-managed essential hypertension, even when their blood pressure is controlled [32].

Using a discovery phase and a validation phase in an observational cohort of 174 hypertension subjects and controls, we found a consistent downregulation of five miRNAs (miR-210-3p, miR-361-3p, miR-362-5p, miR-378a-5p, miR-501-5p) in hypertension subjects as compared with control subjects. Increased levels of hypoxia-induced miR-210-3p were previously linked to pregnancy hypertension [33], pulmonary arterial hypertension in mice [34], mitochondrial dysfunction, myocardial infarction [35], and atrial fibrillation [36]. In addition, Virga et al., 2021, identified miR-210-3p as a non-genetic immunoregulator of macrophage metabolism and inflammatory responses [37].

Several active and selective mechanisms for loading miRNAs into extracellular vesicles suggest that exosomal miRNAs hold promise in contributing to the development of hypertension [17]. Past evidence has demonstrated that exosomal miRNAs can regulate both the deleterious and beneficial pathways of RAAS, such as the angiotensin-converting enzyme (ACE)/Ang II pathway and the ACE2/Mas pathway, respectively. In vitro and in vivo studies have demonstrated that the overexpression of miR-155-5p reduces blood pressure, vascular remodeling, and vascular proliferation by directly decreasing the levels of ACE and, subsequently, Ang II [17]. Exosomal miRNAs can also regulate ACE2, which counterbalances the actions of ACE by degrading its catalytic product, Ang II, into the beneficial heptapeptide Ang-(1-7) [17].

In our discovery cohort, we also observed that miR-361-3p was significantly downregulated in hypertension subjects receiving antihypertensive drugs as compared with the untreated subjects. Since these findings were not confirmed in the validation cohort, they need to be taken with caution given the small and unbalanced sample size of untreated and treated hypertension groups. Elsewhere, Nebivolol, a third-generation, highly selective β1-adrenergic receptor blocker, attenuated the decrease in miR-133a [38]. Several other miRNAs, miR-19a, miR-101, and let-7e, were shown to target genes related to β-blocker pharmacodynamics [39]. Together, these observations suggest that miRNAs may serve as indicators of treatment response and may be useful in companion diagnostics in order to follow medication benefits. Two miRNAs, miR-21 and miR-92, decreased significantly in heart-failure patients with preserved ejection fraction (HFpEF) after three months of empagliflozin treatment, with no notable differences in patients treated with metformin or insulin [40]. These findings highlight the regulation of certain miRNAs related to endothelial function in frail HFpEF patients with diabetes and their response to SGLT2 inhibition [40]. This could serve as valuable information for clinical practice, offering new disease biomarkers and insights into treatment response. MiR-361-5p and miR-362-5p were downregulated in salt-sensitive hypertension patients (ChiCTR-EOC-16009980) from the Beijing population, suggesting a potential protective effect [41]. MiR-362-5p also regulates cell proliferation and apoptosis by targeting glutathione–disulfide reductase in the placenta of women with gestational diabetes mellitus [42]. In addition, miR-361-5p, together with the frequency of sauce and poultry consumption, was selected in a stepwise logistic regression model allowing for the differentiation of salt resistance from salt-sensitive hypertension [41]. Detailed mechanistic insights and causal relationships between miRNA signatures, hypertension subtypes, and evolution and the effect of antihypertensive drugs need to be elucidated in future studies.

In the tested ML model, SVM was the most accurate in discriminating hypertension subjects from control subjects. SVM is a machine learning algorithm used for classification and regression tasks. It is particularly well suited for tasks that involve pattern recognition and data analysis. The fundamental concept behind SVM is to find a hyperplane that best separates different classes of data points in a way that maximizes the margin between the classes. This makes SVM a powerful tool for classification problems, as it aims to identify the most effective boundary or decision boundary between different groups of data. SVM is notably one of the most frequently used ML models because of the following characteristics: (1) it is suitable for classifying complex data, (2) is able to minimize structural risk, and (3) can achieve similar results with different kernel functions like artificial neural networks [43]. In another study, an SVM-based method including 13 anthropometric factors associated with hypertension demonstrated superior performance in comparison with the backpropagation neural network method [44]. However, the study did not consider any single or multi-omics factors associated with hypertension.

Recently, the ENS@T-HT Horizon2020 consortium investigated the ML integration of multi-omics data including 179 plasma miRNAs for the stratification of arterial hypertension. The consortium reported an improved discriminatory power in comparison with single-omics data analysis and was able to discriminate different forms of endocrine hypertension from primary hypertension with high sensitivity and specificity [24]. This study demonstrated that a combination of miRNAs (miR-15a-5p) and small metabolite features (C9 and PC ae C38:1) shows the most discriminating power for all hypertension types of combinations. Suzuki et al. (2021) reported lower levels of miR-126, miR-221, and miR-222 in the blood, which were significantly linked to higher blood pressure and the development of hypertension in new-onset patients [45]. The odds ratios adjusted for confounding factors indicated that, for each one-unit increase in the serum levels of these miRNAs, the risk of new-onset hypertension decreased. This suggests that these circulating miRNAs could serve as potential biomarkers for predicting hypertension [45].

Our SVM model included two miRNAs, miR-361-3p and miR-501-5p, which have not been previously linked to essential hypertension. MiR-361-3p levels were decreased in the plasma of pulmonary arterial hypertension patients [46]. The overexpression of miR-361-3p alleviates cerebral ischemia–reperfusion injuries in mice [47]. MiR-378a-5p is involved in the pathophysiology of cerebrovascular injuries and in various biological processes, including metabolic pathways, mitochondrial energy homeostasis, muscle development, differentiation, and regeneration [48]. MiR-378a-5p was significantly overexpressed in an in vitro model of injury-induced neuronal apoptosis and inhibited cell proliferation through the regulation of the CAMKK2/AMPK pathway [49]. Silencing of the transcription factor Forkhead box O1 (FoxO1) via the lncRNA GAS5/miR-378a-5p/Hspa5 axis in mice significantly improved neurological function recovery in intracerebral hemorrhage, which is the most devastating stroke subtype [50]. Since hypertension is associated with over 50% of ischemic and 70% of hemorrhagic strokes and triggers a 10% increase in the risk of recurrent cerebrovascular events [51], new insights into the role of miR-361-3p and miR-378a-5p in hypertension-associated cerebrovascular pathophysiology may provide key targets to prevent chronic cerebrovascular disease. An emerging body of evidence suggests that miRNAs may be involved in the regulation of the cerebrovascular system, a research axis that deserves attention [52].

Increased levels of miR-501-5p were observed in patients with chronic thromboembolic pulmonary hypertension [53] and in overweight and low-grade obese children and adolescents [54]. Contrary to this last study, miR-501-5p was significantly downregulated in hypertension patients in our study. Overweight and obesity are frequently reported to be associated with hypertension and diabetes mellitus. In the present study, BMI was positively correlated with SBP (r = 0.47, *p* < 0.0001) and DBP (r = 0.51, *p* < 0.001). However, miR-501-5p showed weak negative correlations with BMI (r = −0.19, *p* = 0.01), SBP (r = −0.21, *p* = 0.006), and DBP (r = −0.34, *p* < 0.0001). The molecular mechanisms of miR-501-5p’s association with metabolic disorders need to be further investigated.

Study strengths: We used an approach using a discovery phase and a validation phase in independent groups of subjects, which allowed us to identify confirmed associations between miRNAs and hypertension. The discovery phase was well powered with two groups of 30 age- and sex-matched subjects enrolled in small RNA sequencing experiments. Various ML approaches were tested, leading to the selection of SVM as the most powerful model.

Study limitations: The study cohort had a relatively limited sample size, with a total of 174 subjects. This especially limits the strength of model building using ML approaches and may lead to over-fitting and the possible overestimation of effect sizes despite extensive cross-validation. Even though we used an ML pipeline with test and training sets, the accuracy of the SVM classifier requires extensive validation in larger independent datasets. The association between the two miRNAs included in the model (miR-361-3p and miR-501-5p) and hypertension remains to be functionally characterized. Finally, the ML model’s performance must be rigorously validated in diverse patient populations to assess its accuracy, sensitivity, specificity, and generalizability. This process may require extensive clinical trials and real-world testing.

There are inherent challenges to incorporating an ML model based on miRNAs into clinical practice: (1) Data collection and standardization—gathering and standardizing miRNA data from a diverse patient population might be potentially complex. Ensuring data quality, accuracy, and consistency is crucial for reliable model performance. (2) Implementing the model within existing clinical workflows requires seamless integration with electronic health records, diagnostic equipment, and other systems. This may require modifications to ensure efficient use. (3) Patients and healthcare providers must be comfortable with the model’s use. Education and training may be necessary to ensure both groups understand and accept the technology. (4) Assessing the cost-effectiveness of implementing the model is essential, as healthcare resources are finite. Evaluating the model’s impact on patient outcomes and healthcare costs is crucial prior to its integration into routine clinical practice.

## 4. Perspectives

Our study not only offers insight into the biomarker potential of miRNAs in essential hypertension but also suggests that ML-based modeling represents a promising strategy for hypertension risk assessment.

In the long term, the deep functional characterization of the role of miRNAs in hypertension may reveal novel disease mechanisms and highlight putative drug targets for a more effective prevention and hypertension treatment. MiRNAs may help both in the primary and secondary prevention of hypertension, which could contribute to reducing the socioeconomic burden of hypertension. The fast progress of high-throughput experimental and computational technologies will facilitate the wider use of ML-based techniques to identify diagnostic biomarkers and improve our understanding of complex diseases such as hypertension.

## 5. Methods

### 5.1. Study Design and Procedures

In the present observational case–control study, patients with essential hypertension (n = 97) and controls (n = 96) were recruited from the Department of Internal Medicine at a specialized hospital with a polyclinic, the “Plava Medical Group”, in Tuzla, Bosnia and Herzegovina. Information regarding participant recruitment is described in the Supplementary Material. The study was conducted in accordance with the Declaration of Helsinki and has been authorized by the Research Ethics Committee of the University of Tuzla under reference decision number 03/7-1122-1-2/20 from 21 February 2020. Written informed consent was obtained directly from all involved subjects. The transfer of blood samples and associated data from Bosnia and Herzegovina to Luxembourg was performed in accordance with the Material Transfer Agreement entered between the University of Tuzla, Bosnia and Herzegovina, and the Luxembourg Institute of Health (LIH), Luxembourg, in June 2020.

### 5.2. Whole Blood Collection and RNA Isolation

Blood samples were collected as per the usual protocol for standard clinical biochemistry procedures from individuals in a stable state, commonly after an overnight fast and in the early morning. At the time of enrolment of study participants, 2.5 mL of whole blood was withdrawn via an arterial catheter into PAXgene™ RNA tubes (BD Biosciences, Erembodegem, Belgium) and stored at −20 °C until the transfer for further processing at the LIH. Shipment of samples was conducted on dry ice. RNAs were extracted from blood samples with the PAXgene™ Blood miRNA Kit (Qiagen, Venlo, The Netherlands) according to the manufacturer’s instructions. RNA quantity and quality were determined via optical density using a NanoDrop spectrophotometer and a fragment analyzer (Agilent Technologies, Diegem, Belgium). A sample quality check was performed according to established standards [55,56,57], and 19 samples were discarded from the study because of a lack of a recommended volume of blood in PAXgene RNA blood tubes or low RNA quality (RNA integrity number < 7) and quantity (<20 ng/mL). Therefore, RNA samples from 174 participants (89 hypertension patients and 85 controls) were available for the present study.

### 5.3. Small RNA Sequencing

A discovery phase using small RNA sequencing was performed on samples from 30 age- (±2 years) and sex-matched hypertension patients and 30 controls. A case–control-matching procedure was used to randomly match hypertension patients and controls based on age (maximum allowable difference, ±2 years) and sex (exact match) by using the MedCalc software v20.23. All RNA samples used for small RNA sequencing matched the following quality and quantity criteria: A260/280 value between 1.8 and 2.0, RQN > 7, and concentrations >20 ng/μL. cDNA libraries were generated from 100 ng of RNA with the QIAseq miRNA Library Kit (Qiagen) according to the manufacturer’s guidelines. A unique molecular index was integrated during the reverse transcription process, and libraries were cleaned and sizes selected using a magnetic bead-based method. Purified libraries were quantified with a Qubit^®^ 4 Fluorometer (Thermo Fisher Scientific, Merelbeke, Belgium) and validated with an Agilent 2100 Bioanalyzer (Agilent Technologies). The 60 samples were sequenced using an Illumina NovaSeq 6000 sequencing system (Illumina, Eindhoven, The Netherlands). Library construction and sequencing were performed on the LuxGen platform in Luxembourg.

After trimming the adapters and performing quality control on the small RNA-seq data using the FastQC tool (https://www.bioinformatics.babraham.ac.uk/projects/fastqc/, accessed on 4 October 2021), sequencing reads were mapped to reference miRNA sequences from miRBase 22.1. Then, differentially expressed miRNAs were identified after DESeq2 normalization. Only miRNAs detected with at least ten reads in at least half the samples from the hypertension or control group were included in differential expression analysis, which was based on negative binomial distribution using the DESeq2 R package. MiRNAs with a Benjamini–Hochberg false discovery rate (FDR) < 0.05 were considered significantly differentially expressed.

We generated a volcano plot using ggplot to show the –log10 *p*-values versus log2 fold change of the miRNAs. We used a t-distributed stochastic neighbor embedding (tSNE) plot to visualize sample distribution with the normalized counts of differentially expressed miRNAs. Cluster analysis for differentially expressed miRNAs was performed using the pheatmap R package (https://CRAN.R-project.org/package=pheatmap, accessed on 5 October 2021) with Euclidean distance.

### 5.4. Real-Time Quantitative Polymerase Chain Reaction (RT-qPCR)

Expression levels of selected miRNAs were assessed using RT-qPCR, first in the discovery cohort (n = 60) and second in the validation cohort (n = 114). In total, 100 ng from each sample was reverse transcribed to generate cDNA using the miRCURY LNA RT Kit (Qiagen) in accordance with the manufacturer’s instructions. The resulting cDNAs were diluted 6 times before qPCR, which was carried out in a total reaction volume of 10.0 µL, including 3.0 µL of template cDNA, 1 µL of primers (individual miRCURY LNA miRNA PCR Assays, Qiagen, Supplementary Table S1), 1µL of dH_2_O, and 5.0 µL of 2x miRCURY LNA SYBR^®^ Green (Qiagen). Each PCR plate contained an internal standard calibrator to correct for inter-plate variability, appropriate negative controls (no template control (NTC), and no reverse transcriptase control (NRT) in order to check for potential master mix or sample contamination. qPCR was performed using a CFX96 thermocycler (Bio-Rad, Temse, Belgium). miRNA levels are expressed as the number of miRNA copies per ng of RNA, determined according to standard curves as described previously [30] (Supplementary Material).

### 5.5. Machine Learning Model Selection and Classification

The flowchart of the machine learning model selection is presented in Supplementary Figure S1. We excluded 4 participants who had missing values in hypertension family history to avoid the bias introduced by data imputation. We randomly extracted 20% of the complete data from the non-sequenced samples as a test dataset to evaluate the classification models. The continuous variables were transformed using the cube root method and the categorical variables were transformed using one-hot encoding. We performed feature selection based on 7 clinical variables (age, sex, BMI, alcohol use, current smoker, former smoker, and hypertension family history) and 5 validated miRNAs with recursive feature elimination. We found the optimal estimator and the optimal number features with a 10-fold cross-validation (10CV) from different estimators, including random forest (RF), logistic regression (Logit), linear support vector machine (LinearSVC), and extreme gradient boosting (XGBoost, XGB) with default parameters. The estimator and the number of features, which provided the highest balanced accuracy, were used for the feature selection. Given the optimal estimator and the number of features, we selected the features using the sub-training sets split by a 10-fold cross-validator applied to the training dataset. We kept the features appearing in at least 8 sub-training sets. Using the training data of the selected features, we finetuned the hyperparameters of different ML models, i.e., random forest (RF), support vector machine (SVM), multi-layer perceptron (MLP), extreme gradient boosting (XGBoost, XGB), k-nearest neighbor’s (kNN), and logistic regression (Logit), with 2 repeated 10CV. The model with the hyperparameters—which provided the highest mean balanced accuracy in the validation datasets and the difference between the training and validation scores that was no more than 10% of the higher score—was considered the final model.

We evaluated the final model using the test dataset and the whole dataset with Leave-One-Out Cross-Validation (LOOCV). We used the area under the receiver operating characteristic curve (AUC), balanced accuracy, F1 score, precision, sensitivity (recall), and specificity to quantify the quality of classification. The ML analysis was performed using Python 3.8 and scikit-learn 0.24.2 (https://scikit-learn.org/0.24/, accessed on 25 October 2021)

### 5.6. Statistical Analysis

Data were analyzed using GraphPad Prism version 9.0.0. The Shapiro–Wilk test was employed to determine whether the data were normally distributed based on probability thresholds of >0.1. Continuous variables were expressed as mean and standard deviation (SD) when normally distributed. Median and 25th and 75th percentiles were used for skewed variables. Categorical variables were reported as counts and percentages. When comparing groups, a *t*-test or the Mann–Whitney Rank Sum Test was used for continuous variables, and the chi-square test was used for categorical variables. Pearson’s correlation coefficient (r) and *p*-value were calculated to explore the association between small RNA sequencing data and RT-qPCR data. A *p*-value < 0.05 was used as the significance threshold.

## 6. Conclusions

The identification of biomarkers for complex multifactorial disorders such as essential hypertension is an important challenge to overcome in the application of individualized risk assessment strategies and tailored treatments. We propose an ML model based on clinical features and miRNAs to address this challenge, which may allow for more precise and personalized approaches to hypertension by identifying individuals at risk of hypertension at an earlier stage, facilitating timely interventions and lifestyle adjustments to mitigate these risks. This proactive approach may empower patients to undertake preventive measures and make the necessary lifestyle changes to reduce the risk of hypertension and related cardiovascular diseases. In addition, by using such an ML model, clinicians may optimize therapeutic approaches and medications for hypertensive patients, potentially enhancing treatment efficacy while minimizing side effects. Given that hypertension stands as a prominent risk factor for cardiovascular diseases, the model’s ability to enable early detection and optimized management may have the potential to significantly reduce the healthcare system’s burden, improve overall health, and extend life expectancy across the population.

## Figures and Tables

**Table 1:** Clinical and demographic features of the study population.

Clinical Characteristics	Discovery Cohort (n = 60)			Validation Cohort (n = 114)		
	Control Group (n = 30)	Hypertension Group (n = 30)	*p*	Control Group (n = 55)	Hypertension Group (n = 59)	*p*
Mean age	45.6 ± 13.167	45.7 ± 13.02	0.955	43.91 ± 15.44	48.57 ± 12.72	0.079
Gender						
Males, *N* (%)	15 (50%)	15 (50%)	-	24 (43.64%)	35 (59.32%)	0.133
Females, *N* (%)	15 (50%)	15 (50%)	-	31 (56.36%)	24 (40.68%)	
BMI (kg/m^2^)	25.25 ± 2.238	30.52 ± 3.377	0.0001	25.27 ± 2.95	28.93 ± 3.14	0.0001
Blood pressure						
Systolic blood pressure (mmHg)	120.43 ± 5.67	147.8 ± 13.67	0.0001	122.2 ± 7.91	150.5 ± 14.39	0.0001
Diastolic blood pressure (mmHg)	77.57 ± 6.24	98.33 ± 6.73	0.0001	78.0 ± 6.55	97.42 ± 5.38	0.0001
Hypertension treatment						
Untreated, *N* (%)	-	6 (20%)	-	-	14 (23.73%)	-
Treated, *N* (%)		24 (80%)	-	-	45 (76.27%)	
Anti-hypertension therapy						
No therapy, *N* (%)	-	6 (20%)	-	-	14 (23.73%)	-
Monotherapy, *N* (%)	-	19 (63.33%)	-	-	37 (62.71%)	
Combined therapy, *N* (%)		5 (16.67%)	-	-	8 (13.56%)	
Complications caused by hypertension						
No complications, *N* (%)	-	27 (90%)	-	-	54 (91.53%)	-
With complications, *N* (%)		3 (10%)	-	-	5 (8.47%)	
Diabetes mellitus						
Non-diabetic patients, *N* (%)	-	-	-	-	50 (84.75%)	-
Diabetic patients, *N* (%)	-	-	-	-	8 (13.56%)	-
Hospitalization due to uncontrolled hypertension, *N* (%)	-	-	-	-	1 (1.69%)	-
Smoking status						
Current smokers, *N* (%)	5 (16.66%)	17 (56.66%)	0.005	13 (23.64%)	27 (45.76%)	0.003
Former smokers, *N* (%)	6 (20.00%)	3 (10.00%)		4 (7.27%)	10 (16.95%)	
Non-smokers, *N* (%)	19 (63.33%)	10 (33.33%		38 (69.09%)	22 (37.29%)	
Alcohol consumption, *N* (%)	5 (16.66%)	12 (40.00%)	0.084	39 (70.91%)	25 (42.37%)	0.002
Hypertension family history						
Positive family history	13 (43.33%)	24 (80%)	0.010	16 (29.09%)	45 (76.27%)	0.999
Negative family history	15 (50%)	6 (20%		37 (67.27%)	14 (23.73%)	
Unknown family history	2 (6.67%)	0		2 (3.64%)	0	

**Table 2:** Seven differentially expressed miRNAs between hypertension and control subjects in the discovery cohort.

	Mean of Normalized Counts				
miRNA	Control Group (n = 30)	Hypertension Group (n = 30)	Log2 FC	*p*	FDR
hsa-miR-186-5p	9620	7314	−0.410	0.0013	0.043
hsa-miR-210-3p	130	81	−0.653	0.0012	0.041
hsa-miR-361-3p	3917	3113	−0.344	0.0002	0.028
hsa-miR-362-5p	450	317	−0.494	0.0002	0.028
hsa-miR-378a-5p	349	248	−0.515	0.00005	0.018
hsa-miR-501-5p	26	18	−0.503	0.0008	0.038
hsa-miR-769-5p	47	30	−0.616	0.00003	0.015

FC indicates fold change; FDR—Benjamini–Hochberg false discovery rate.

**Table 3:** Performance metrics of the SVM model using the test dataset and LOOCV on the whole dataset.

	AUC	Balanced Accuracy	F1 (Hypertension)	Precision (Hypertension)	Sensitivity (Hypertension)	Specificity (Hypertension)
Test dataset	0.90	0.87	0.87	0.91	0.83	0.91
LOOCV	0.89	0.83	0.83	0.87	0.80	0.86

LOOCV indicates Leave-One-Out Cross-Validation; AUC—area under the receiver operating characteristic curve.

**Table 4:** Performance metrics of the SVM model only with clinical variables using the test dataset and LOOCV across the whole dataset.

	AUC	Balanced Accuracy	F1 (Hypertension)	Precision (Hypertension)	Sensitivity (Hypertension)	Specificity (Hypertension)
Test dataset	0.89	0.87	0.87	0.91	0.83	0.91
LOOCV	0.87	0.79	0.80	0.81	0.79	0.80

## Data Availability

Data Availability Statements are available in the “MDPI Research Data Policies” at https://www.mdpi.com/ethics, accessed on 25 October 2021.

## Supplementary Materials

The following supporting information can be downloaded at https://www.mdpi.com/article/10.3390/ncrna9060064/s1: Table S1: List of miCURY LNA miRNA PCR assays used in the present study. Table S2: Linear regression data from qRT-PCR for miRNAs absolute quantification. Table S3: Feature selection in the ten sub-training sets. Table S4: Hyperparameter tuning of six classifiers. Figure S1: Machine learning workflow. Figure S2: Correlation between miRNA expression determined with RT-qPCR and small RNA sequencing (RNAseq) data. Figure S3: Effect of medication on miRNA levels in the discovery cohort. Figure S4: Effect of medication on miRNA levels in the validation cohort. Figure S5: Correlation between miRNA expression levels and clinical variables. Figure S6: Optimal number of features (highest balanced accuracy) from logistic regression (Logit), random forest (RF), linear support vector machine (LinearSVC), and XGBoost (XGB). Figure S7: Classification accuracy and sensitivity between hypertension patients and controls. Figure S8: Evaluation scores of the SVM model using 10 repeated 10CVs across the whole dataset of 7 selected features or 5 clinical features only.

## Abbreviations

10CV	10-fold cross-validation
AH	Arterial hypertension
AUC	Area under the receiver operating characteristic curve
AI	Artificial intelligence
BP	Blood pressure
ECG	Electrocardiogram
HFpEF	Heart failure with preserved Ejection Fraction
XGBoost, XGB	Extreme gradient boosting
kNN	k-nearest neighbor’s
LOOCV	Leave-One-Out Cross-Validation
LinearSVC	Linear support vector machine
Logit	Logistic regression
ML	Machine learning
miRNAs	MicroRNAs
MLP	Multi-layer perceptron
ncRNA	Non-coding RNA
NRT	No reverse transcriptase control
NTC	No template control
RF	Random forest
RT-qPCR	Real-time quantitative polymerase chain reaction
SVM	Support vector machine
t-SNE	t-distributed stochastic neighbor embedding
