# Supplementary material for: A Machine Learning Model Based on microRNAs for the Diagnosis of Essential Hypertension
Source: Noncoding RNA. 2023 Oct 25;9(6):64. doi: 10.3390/ncrna9060064 (PMC10660456; doi:10.3390/ncrna9060064)
Supplement: Supplementary file 1 [file ncrna-09-00064-s001.zip › ncrna-2638339-supplementary.pdf]

A machine learning model based on microRNAs for the diagnosis of essential hypertension

Amela Jusic, PhD[1,2], Inela Junuzovic, MSc[3], Ahmed Hujdurovic, MD[3], Lu Zhang, MSc[4], Mélanie Vausort, MSc[1], Yvan Devaux, PhD[1]

[1]Cardiovascular Research Unit, Department of Precision Health, Luxembourg Institute of Health, L-1445 Strassen, Luxembourg.

[2]Haya Therapeutics, 1066 Epalinges, Switzerland

[3]Department of Internal Medicine, Medical Center "*Plava Poliklinika*", 3rd *Tuzlanska Brigade* No. 7, 75000, Tuzla, Bosnia and Herzegovina.

[4]Bioinformatics Platform, Luxembourg Institute of Health, L-1445 Strassen, Luxembourg.

Address correspondence to:

Yvan Devaux, Cardiovascular Research Unit, Luxembourg Institute of Health, 1A-B rue Edison, L-1445 Strassen, Luxembourg. Tel: +352 26970300. Fax: +352 26970396. Email: yvan.devaux@lih.lu

Supplementary Material

Study subjects

Demographic, anthropometric, health history, dietary and smoking status data were collected from 193 eligible subjects by using a standardized questionnaire specifically developed for the present study. Physical examination involved blood pressure (BP) measurement according to the ESC/ESH Guidelines for the management of arterial hypertension[44], body weight and height.

Inclusion criteria: 1) subjects older than 18 years, 2) BP<140/90 mm Hg (normotensive subjects), BP >140/90 mm Hg (hypertensive subjects, divided into two subgroups: a) screen-detected and untreated hypertension patients and b) patients with known essential hypertension treated with anti-hypertensive medications).

Exclusion criteria: 1) patients with secondary hypertension (history of kidney diseases, adrenal disease, endocrine disorders (hyperparathyroidism**,** thyroid problems)**,** obstructive sleep apnea); 2) subjects with the following diseases: coronary heart disease, severe pulmonary insufficiency, rheumatic diseases, severe liver disorders, malignancies or history of mental illness; 3) use of following medicaments: hormonal contraceptives (birth control pills), non-steroidal anti-inflammatory agents, diet pills, stimulants, antidepressants, immune system suppressants, decongestants; 4) pregnant or lactating women.

MicroRNAs absolute quantification

Synthetic miR-186-5p, miR-210-3p, miR-362-5p, miR-378a-5p, miR-501-5p miRCURY LNA miRNA mimics and miR-361-3p miScript miRNA mimic (5nmol; Qiagen, Venlo, The Netherlands) were dissolved in 250µL of nuclease-free water to obtain 20µM stock solutions

($1.204x10^{13}$ copies/µl). Working solutions were prepared from these stock solutions to obtain 10^{10} copies/µl. Serially diluted synthetic miRNAs to final concentrations from 10^{10} copies/µl to 10^{6} copies/µl in two identical replicates of equal volumes were used to generate standard curves. For each standard curve point, 2µL of mimic dilution was reverse transcribed using the miRCURY LNA RT Kit in presence of 25ng of carrier RNA (Qiagen). Negative reverse transcription controls were obtained with 25ng of carrier RNA only. MiRNA-specific quantitative PCRs (miRCURY; Qiagen) were performed with 3µL of cDNA after a 60-fold dilution. Each assay was run in duplicate and a standard curve from 10^{8} to 10^{4} copies per well was produced. The correlation coefficient (R^2), the slope (m) and the Y-axis intercept (b) were determined from miRNA-specific standard curves using the CFX96 Manager 3.1 software (Bio-Rad, Temse, Belgium) and are indicated in Table S1. For each sample, the number of miRNA copies per well of PCR plate was obtained with the formula: $10^{((Cq\ value - b)/m)}$. All Cq values from patient samples were in the range of the standard curves. The number of miRNA copies per ng of RNA used as template was obtained by multiplying this last value with a correction factor of 2 to account for dilutions during the process. Expression levels of miRNAs were finally expressed as number of copies per ng of RNA.

Table S1. List of miCURY LNA miRNA PCR assays used in the present study.

Gene	MIMAT ID	Sequence	GenGlobe ID Qiagen
hsa-miR-186-5p	MIMAT0000456	5'CAAAGAAUUCUCCUUUUGGGCU	YP00206053
hsa-miR-210-3p	MIMAT0000267	5'CUGUGCGUGUGACAGCGGCUGA	YP00204333
hsa-miR-361-3p	MIMAT0004682	5'UCCCCCAGGUGUGAUUCUGAUUU	YP00204008
hsa-miR-362-5p	MIMAT0000705	5'AAUCCUUGGAACCUAGGUGUGAGU	YP00204618
hsa-miR-378a-5p	MIMAT0000731	5'CUCCUGACUCCAGGUCCUGUGU	YP00204347
hsa-miR-501-5p	MIMAT0002872	5'AAUCCUUUGUCCCUGGGUGAGA	YP00204648
hsa-miR-769-5p	MIMAT0003886	5'UGAGACCUCUGGGUUCUGAGCU	YP00204270

Table S2. Linear regression data from qRT-PCR for miRNAs absolute quantification.

miRNAs	R^2	Slope (m)	Y-axis intercept (b)
miR-186-5p	0.993	-3.071	38.587
miR-210-3p	0.999	-3.153	41.518
miR-361-3p	0.995	-3.465	36.586
miR-362-5p	0.996	-3.005	34.188
miR-378a-5p	0.983	-3.071	41.996
miR-501-5p	0.997	-3.529	44.079

Table S3. Feature selection in the ten sub-training sets.

Fold_1	Fold_2	Fold_3	Fold_4	Fold_5	Fold_6	Fold_7	Fold_8	Fold_9	Fold_10
BMI	BMI	BMI	BMI	Age	BMI	BMI	BMI	BMI	BMI
current smoker	current smoker	current smoker	current smoker	BMI	current smoker	current smoker	current smoker	current smoker	current smoker
alcohol use	alcohol use	alcohol use	alcohol use	alcohol use	alcohol use	alcohol use	alcohol use	alcohol use	alcohol use
Sex	Sex	Sex	Sex	Sex	Sex	Sex	Sex	Sex	Sex
hypertension family history	hypertension family history	hypertension family history	hypertension family history	hypertension family history	hypertension family history	hypertension family history	hypertension family history	hypertension family history	hypertension family history
miR-361	miR-361	miR-361	miR-361	miR-361	miR-361	miR-361	miR-361	miR-361	miR-361
miR-501	miR-501	miR-501	miR-501	miR-501	miR-501	miR-501	miR-501	miR-501	miR-501

Table S4. Hyperparameter tuning of six classifiers.

Classifier	Training		Validation		Difference (% training)
	Balanced accuracy	SD	Balanced accuracy	SD	
**RF**	1.000	0.000	0.830	0.081	17.01%
**kNN**	1.000	0.000	0.834	0.096	16.56%
**Logit**	0.838	0.012	0.828	0.083	1.18%
**XGB**	0.969	0.009	0.810	0.079	16.44%
**MLP**	0.847	0.012	0.828	0.081	2.33%
**SVM**	0.878	0.010	0.833	0.093	5.20%

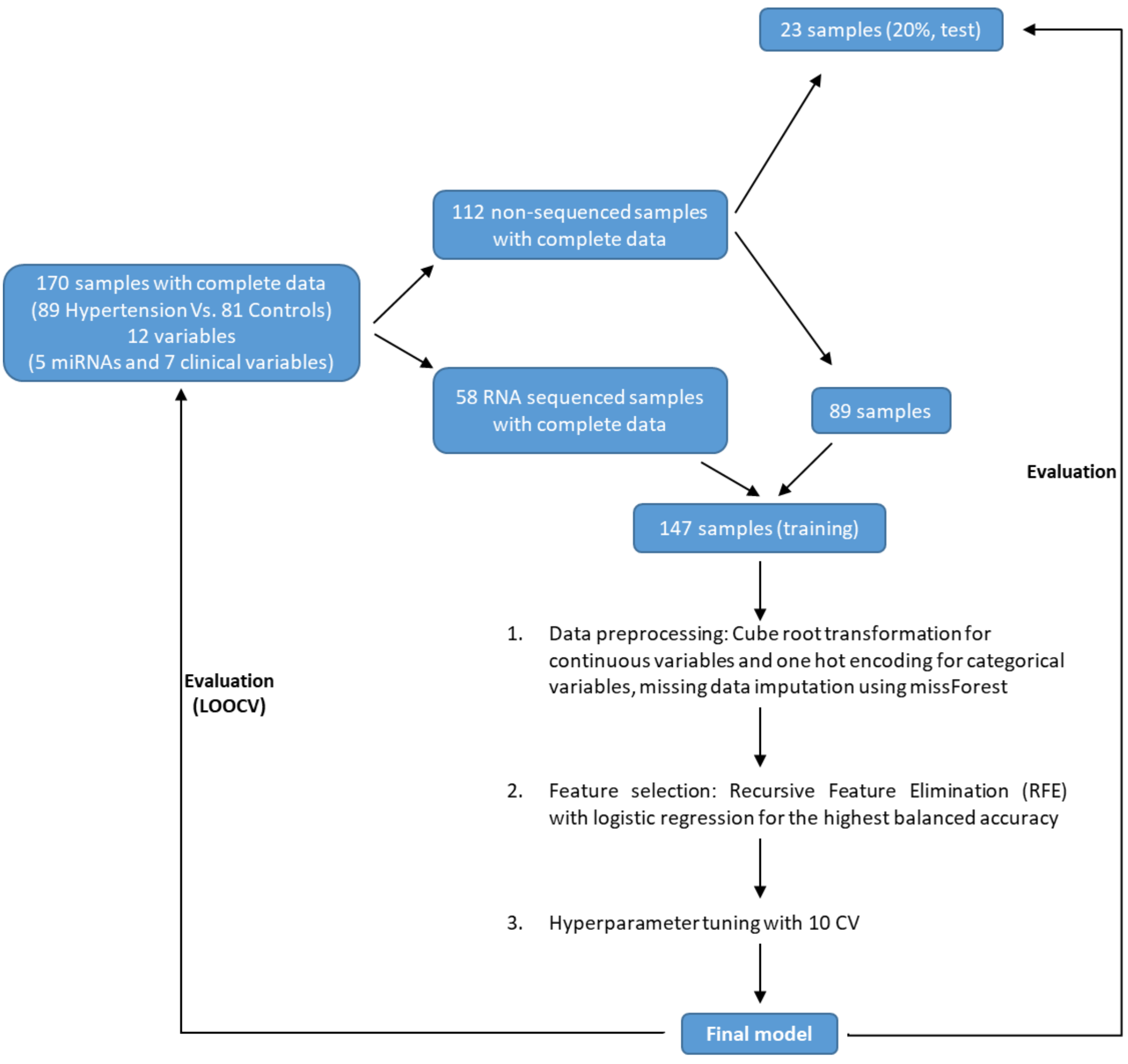

Figure S1. Machine learning workflow.

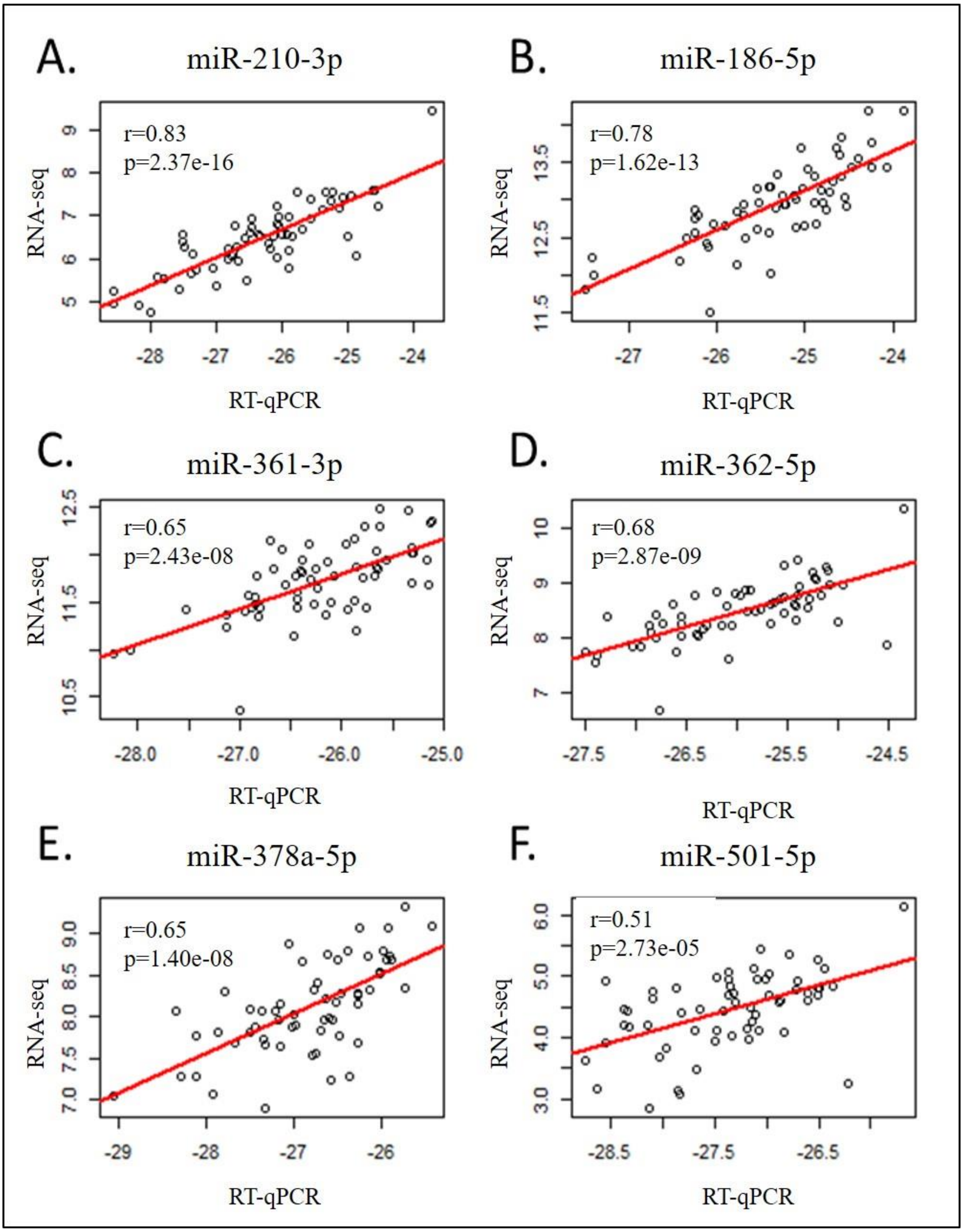

Figure S2. Correlation between miRNAs expression determined by RT-qPCR and small RNA sequencing (RNAseq) data. Pearson's correlation coefficient (r) and p-values are indicated in each plot.

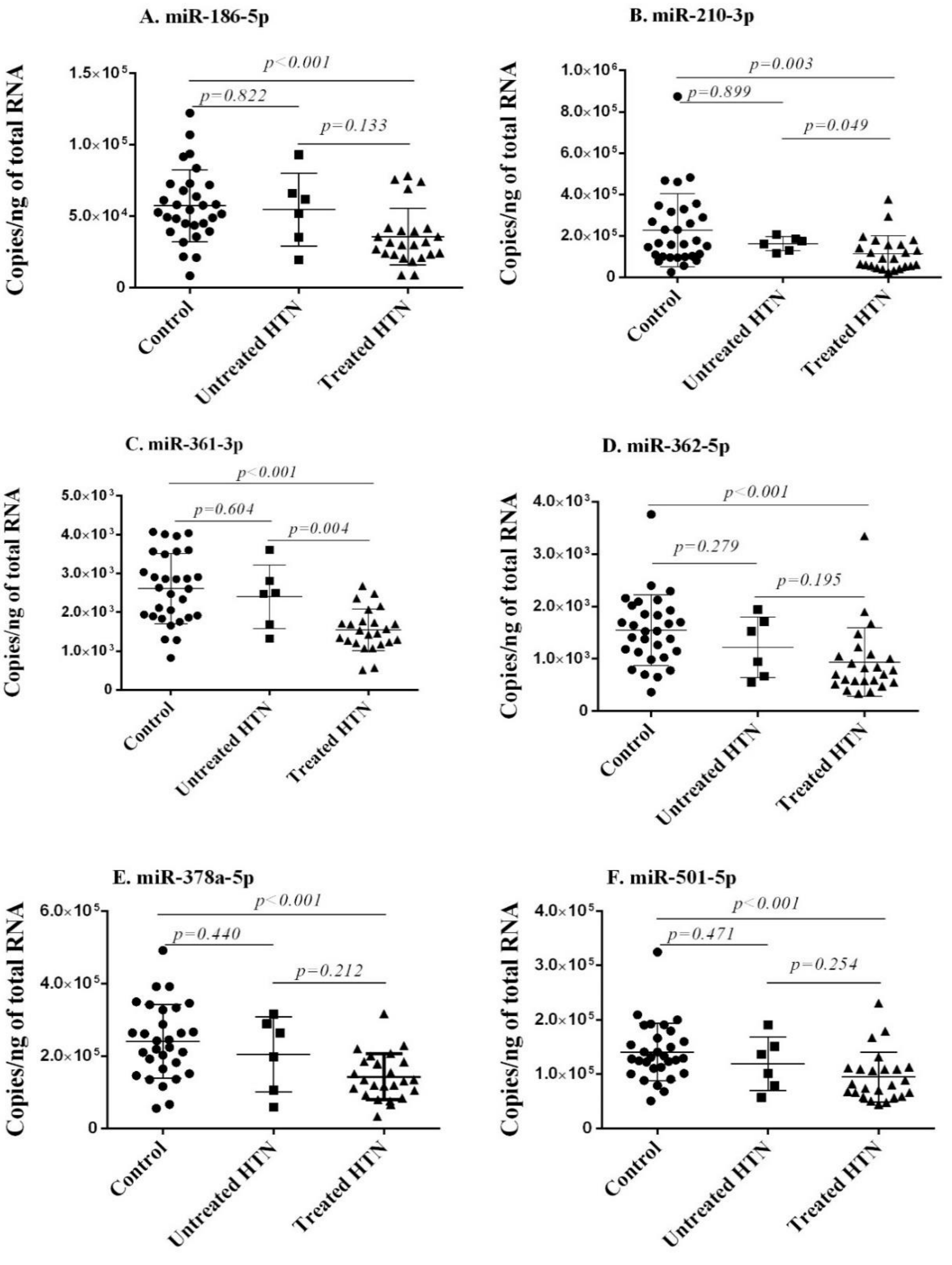

Figure S3. Effect of medication on miRNA levels in the discovery cohort. Scatter plots show the expression levels of 6 miRNAs determined by RT-qPCR in whole blood samples of controls (n=30), untreated hypertension subjects (n=6) and hypertension subjects receiving an anti-hypertensive treatment (n=24). Expression levels of miRNAs are expressed as number of copies per ng of RNA. P-values from ANOVA test (multiple comparisons) are indicated.

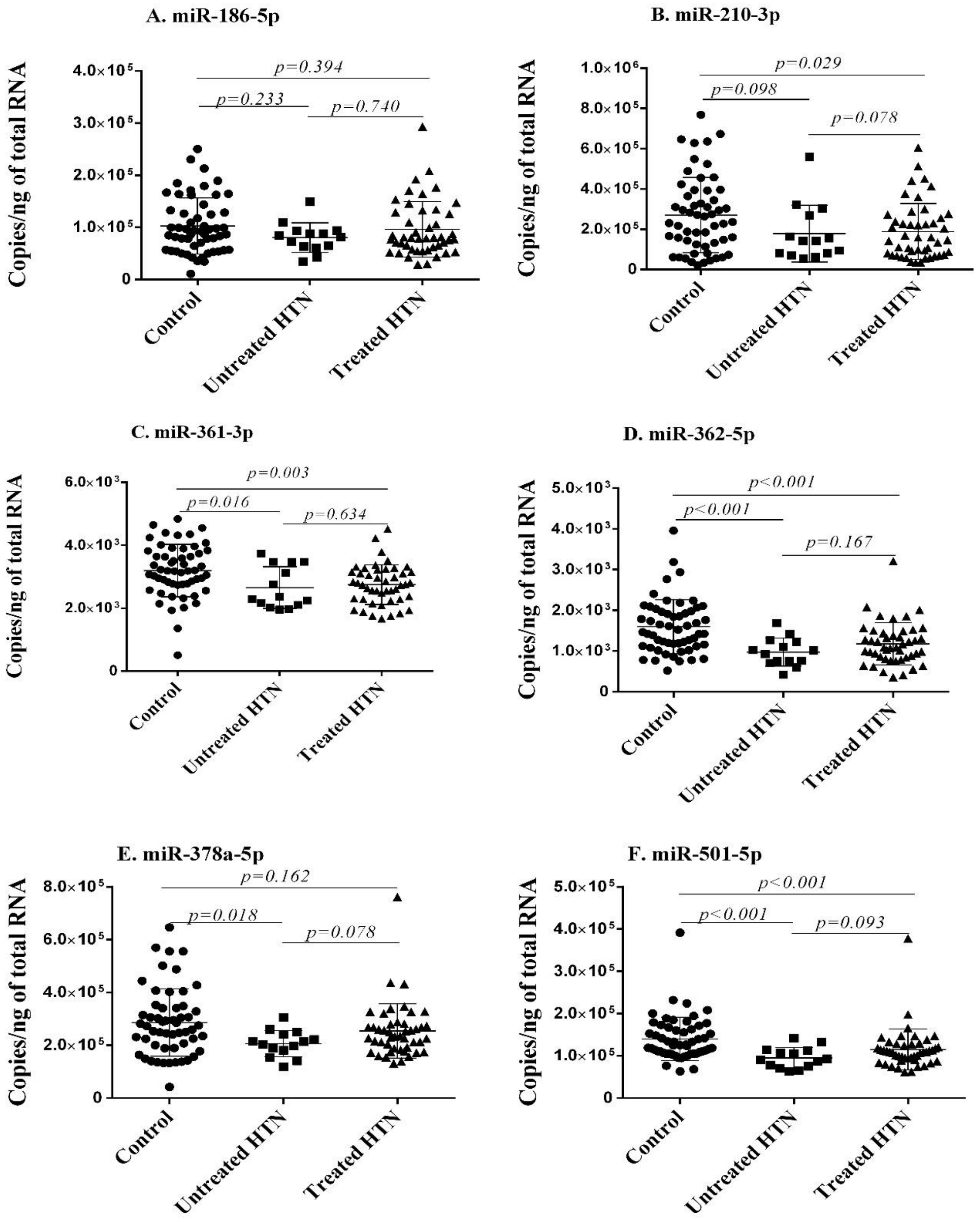

Figure S4. Effect of medication on miRNA levels in the validation cohort. Scatter plots show the expression levels of 6 miRNAs determined by RT-qPCR in whole blood samples of controls (n=55), untreated hypertension subjects (n=14) and hypertension subjects receiving an anti-hypertensive treatment (n=45). Expression levels of miRNAs are expressed as number of copies per ng of RNA. P-values from ANOVA test (multiple comparisons) are indicated.

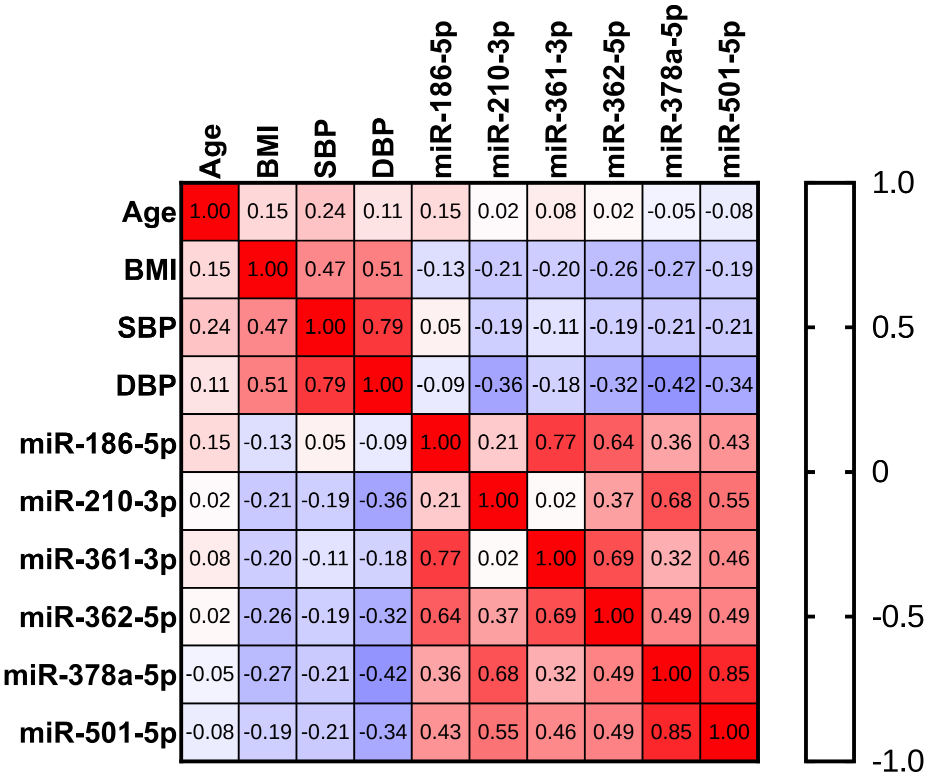

Figure S5. Correlation between miRNA expression levels and clinical variables. Heat-map showing the correlation between the expression levels of 6 miRNAs determined by RT-qPCR in whole blood samples of 55 controls and 59 hypertension subjects. Pearson correlation coefficients are indicated in the squares and as a colour code, red being the highest positive correlation (r=1) and blue being the highest negative correlation (r=-1). BMI - body mass index; BMP - systolic blood pressure; DBP - diastolic blood pressure.

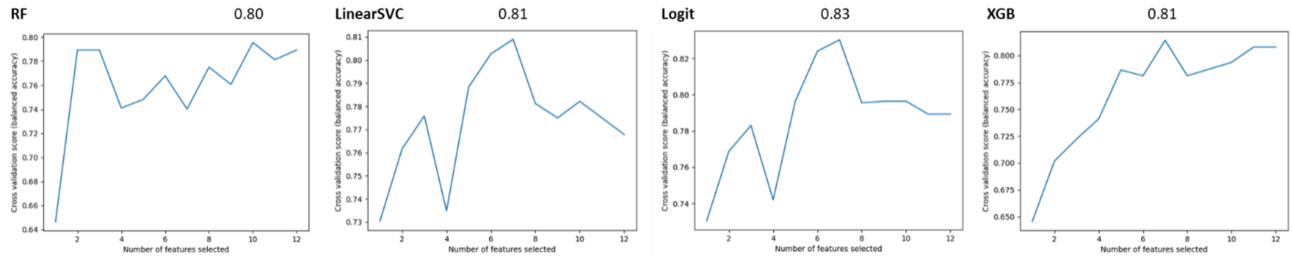

Figure S6. Optimal number of features (highest balanced accuracy) from logistic regression (Logit), random forest (RF), linear support vector machine (LinearSVC) and XGBoost (XGB). The logistic model gave the highest balanced accuracy of 0.83 when using 7 features. So we selected 7 as the optimal number of features. Seven features were selected using recursive feature elimination with logistic regression model in 10 sub-training sets split by a 10CV applied on the training dataset. The features sex, BMI, current smoker, alcohol use, hypertension family history, miR-361-3p and miR-501-5p were selected at least 8 times in the 10 sub-training sets.

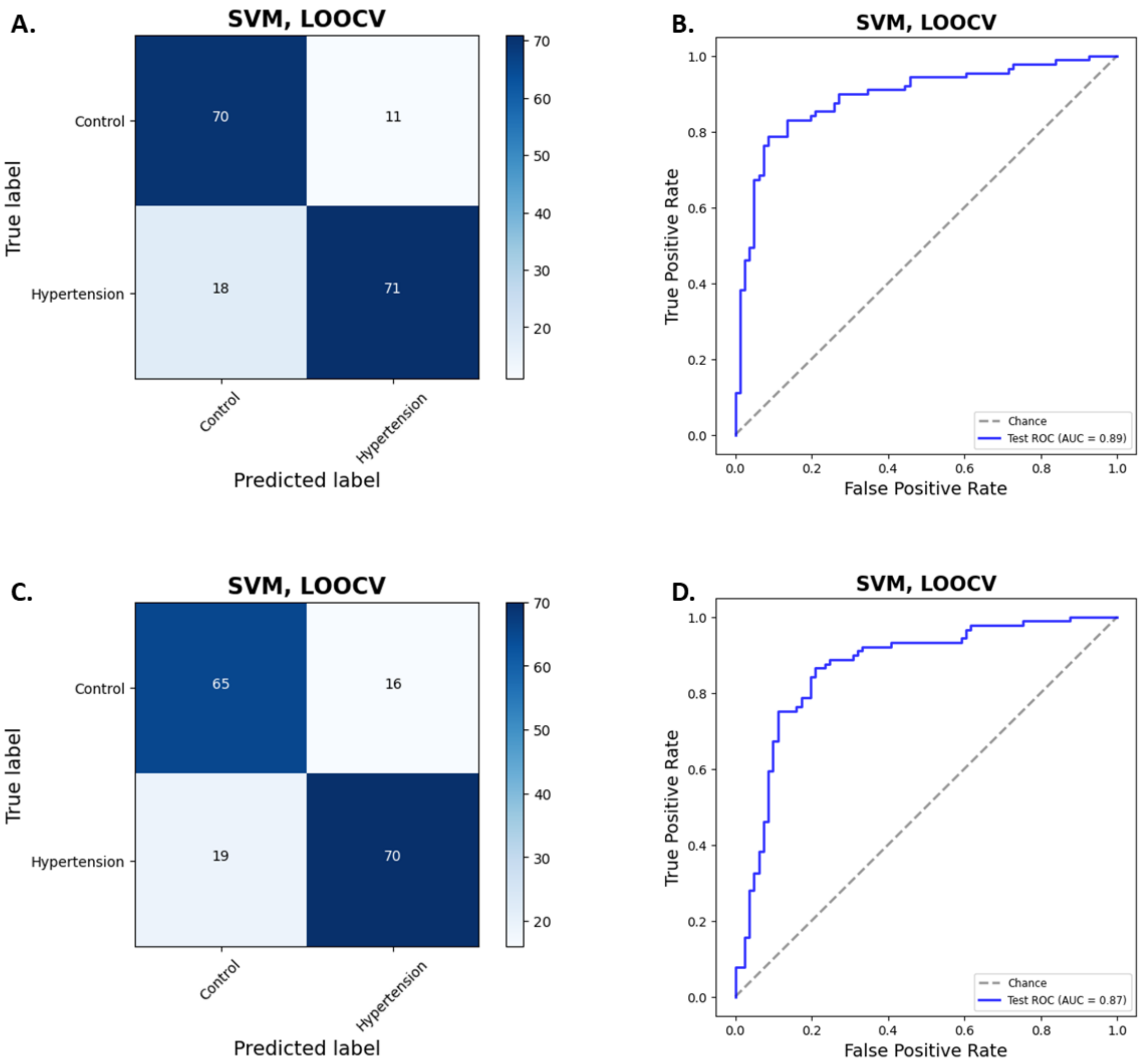

Figure S7. Classification accuracy and sensitivity between hypertension patients and controls. Confusion matrix of the final SVM model using LOOCV across the whole dataset of 170 subjects of 7 selected features (A) or 5 clinical features only (C). The proportion of samples falling into the predicted group (column) and the true group (row) is represented by colour intensity (blue). ROC curve of the final SVM model using LOOCV across the whole dataset with 7 selected features (B) or 5 clinical features only (D). LOOCV - Leave One Out Cross Validation; ROC - receiver operating characteristic; AUC - area under the ROC curve.

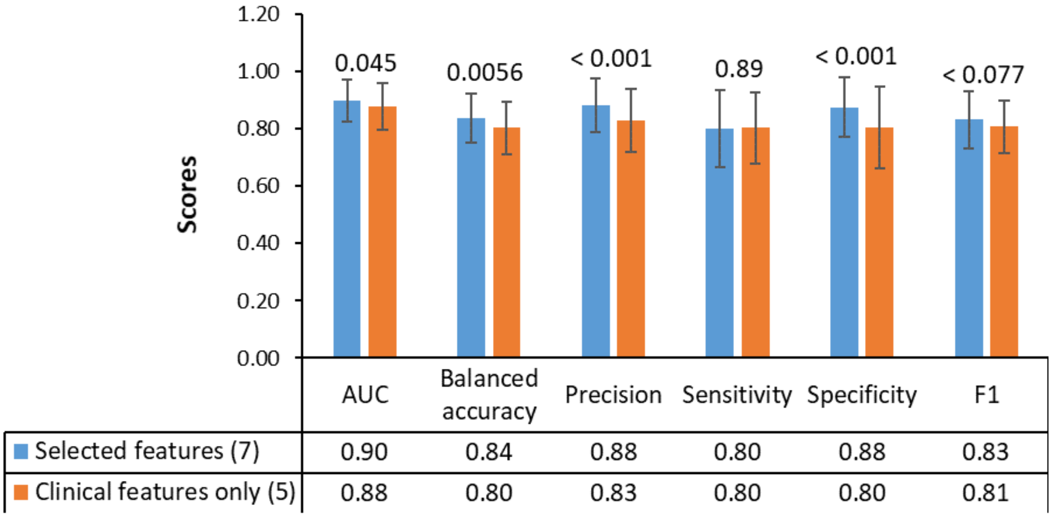

	AUC	Balanced accuracy	Precision	Sensitivity	Specificity	F1
Selected features (7)	0.90	0.84	0.88	0.80	0.88	0.83
Clinical features only (5)	0.88	0.80	0.83	0.80	0.80	0.81

Figure S8. Evaluation scores of the SVM model using 10 repeated 10CV across the whole dataset of 7 selected feature or 5 clinical features only. The numbers above the bars presented the p values from Student's t-test.
